# Impact of *Shigella* infections and inflammation early in life on child growth and school-aged cognitive outcomes: Findings from three birth cohorts over eight years

**DOI:** 10.1371/journal.pntd.0010722

**Published:** 2022-09-23

**Authors:** Elizabeth T. Rogawski McQuade, Rebecca J. Scharf, Erling Svensen, Amber Huggins, Angelina Maphula, Eliwaza Bayo, Ladislaus Blacy, Paula Pamplona E. de Souza, Hilda Costa, Eric R. Houpt, Pascal O. Bessong, Estomih R. Mduma, Aldo A. M. Lima, Richard L. Guerrant

**Affiliations:** 1 Department of Epidemiology, Emory University, Atlanta, Georgia, United States of America; 2 Department of Pediatrics, University of Virginia, Charlottesville, Virginia, United States of America; 3 Haukeland University Hospital, Bergen, Norway; 4 University of Venda, Thohoyandou, South Africa; 5 Haydom Global Health Research Centre, Haydom Lutheran Hospital, Haydom, Tanzania; 6 Universidade Federal do Ceara, Fortaleza, Brazil; 7 Division of Infectious Diseases and International Health, University of Virginia, Charlottesville, Virginia, United States of America; Mohammed Bin Rashid University of Medicine and Health Sciences, UNITED ARAB EMIRATES

## Abstract

**Background:**

*Shigella* infections cause inflammation, which has been hypothesized to mediate the associations between *Shigella* and child development outcomes among children in low-resource settings. We aimed to assess whether early life inflammation and *Shigella* infections affect school-aged growth and cognitive outcomes from 6–8 years of age.

**Methodology/principal findings:**

We conducted follow-up assessments of anthropometry, reasoning skills, and verbal fluency in 451 children at 6–8 years of age in the Brazil, Tanzania, and South Africa sites of MAL-ED, a longitudinal birth cohort study. We estimated the associations between *Shigella* burden and inflammation with linear growth at 2, 5, and 6–8 years of age, and with the cognitive test scores using linear regression and adjusting for potential confounding variables. We also assessed whether inflammation mediated the associations between *Shigella* and school-aged outcomes using a regression-based approach to mediation analysis. A high prevalence of *Shigella* was associated with a 0.32 (95% CI: 0.08, 0.56) z-score lower height-for-age z-score (HAZ) at 6–8 years compared to a low prevalence of *Shigella*. Intestinal inflammation had a smaller association with HAZ at 6–8 years. *Shigella* burden had small and consistently negative associations with cognitive outcomes in Brazil and Tanzania, but not South Africa, and the estimates were not statistically significant. Systemic inflammation was strongly associated with lower verbal fluency scores in Brazil (semantic fluency z-score difference: -0.57, 95% CI: -1.05, -0.10; phonemic fluency z-score difference: -0.48, 95% CI: -0.93, -0.03). There was no evidence that intestinal inflammation mediated the association between *Shigella* and HAZ or cognitive outcomes.

**Conclusions/significance:**

While *Shigella* infections were consistently associated with long-term deficits in linear growth, the estimates of the negative associations between *Shigella* and cognitive outcomes were imprecise and only observed in the Brazil and Tanzania sites. Systemic inflammation was strongly associated with lower semantic and phonemic fluency scores in Brazil only, highlighting the site-specificity of effects.

## Author summary

*Shigella* infections are common among children in low-resource settings and cause inflammation, which may contribute to poor child development outcomes. We studied children from birth to 6–8 years of age in Brazil, Tanzania, and South Africa to assess whether *Shigella* infections and markers of inflammation in the first 2 years of life were associated with child development outcomes at school age, including height and cognitive assessment scores. *Shigella* infections were consistently associated with long-term deficits in linear growth. However, the associations between *Shigella* and cognitive outcomes were smaller, not statistically significant, and inconsistent across the three sites. There was no evidence that inflammation caused by *Shigella* explained the associations between *Shigella* and school-aged outcomes. Systemic inflammation was strongly associated with lower cognitive assessment scores in Brazil but not in the other sites, highlighting the differences in determinants of child development across settings. Quantifying the impact and understanding mechanisms for the effect of early life exposures to *Shigella* on long-term cognitive outcomes remains challenging.

## Introduction

Intestinal inflammation is one of the defining components of environmental enteric dysfunction (EED), a subclinical condition of the gut that is also characterized by dysfunction in the intestinal epithelial barrier, reduced gut immune function, and malabsorption [1]. EED is common among children in low-resource settings and results from chronic environmental exposure to enteric pathogens through inadequate access to safe water and sanitation [2,3]. Several enteric pathogens directly cause inflammation, most notably *Shigella*, which secretes virulence factors that have enterotoxic activity and allow *Shigella* to invade the colonic epithelium [4]. *Shigella*-associated inflammation has been observed even in the absence of other clinical symptoms. *Shigella* detections in both diarrheal and non-diarrheal stools in the first two years of life were associated with increases in a biomarker of intestinal inflammation, myeloperoxidase (MPO), in the 8-site MAL-ED birth cohort study. The association depended on *Shigella* quantity, such that MPO levels were more highly elevated as the quantity of *Shigella* increased [5].

Both enteric pathogens and inflammation have been associated with linear growth faltering among children in low-resource settings. Bacterial and protozoal pathogens were associated with larger decrements in length than viral pathogens in MAL-ED. Compared to other pathogens, *Shigella* was associated with the largest decrements in length-for-age z-score at 2 years of age per log increase in the mean pathogen quantity detected in non-diarrheal stools from 0–2 years [6]. Furthermore, *Shigella* was the only pathogen for which the association with length persisted to 5 years of age, suggesting uniquely sustained impact. Inflammation, as measured by fecal MPO and serum alpha-1-acid glycoprotein (AGP), a marker of systemic inflammation, was associated with reduced linear growth in this study [7], and models suggested that the associations between invasive bacteria and growth were mediated by inflammation [8].

Linear growth is commonly used as a proxy for cognitive function [9], arguably a more public health-relevant outcome, since anthropometry is easier to measure and requires shorter-term follow-up. However, while some mechanistic pathways for effects on growth and cognition may be shared, there could be independent effects of inflammation on cognitive function [10], though the evidence is mixed [11]. Inflammation particularly affects brain development in children [12], and is thought to cause brain damage through the direct effects of inflammatory cytokines [13] and inflammation-related proteins [14,15]. In support of these pathways, fever and biomarkers of inflammation during infancy were associated with reductions in cognitive assessment scores in Dhaka, Bangladesh [16]. Total enteric pathogen burden was weakly associated with cognitive outcomes at 2 years of age [17], and was not significantly associated with cognition at 5 years of age in MAL-ED [18]. However, cognitive effects are likely to be pathogen-specific, as were effects on linear growth [6], given major differences in pathogenesis.

To directly assess whether early life inflammation and exposures to the inflammatory pathogen, *Shigella*, affect long-term child development, we conducted a follow-up study of children at three sites of the MAL-ED birth cohort study to assess impact on linear growth and school-aged cognitive outcomes from 6–8 years of age.

## Methods

### Ethics statement

The study was approved by the Institutional Review Board for Health Sciences Research, University of Virginia, USA (HSR IRB #14595) as well as the respective governmental, local institutional, and collaborating institutional ethical review boards at each site: Committee for Ethics in Research, Universidade Federal do Ceara; National Ethical Research Committee, Health Ministry, Council of National Health (Brazil); Health, Safety and Research Ethics Committee, University of Venda; Department of Health and Social Development, Limpopo Provincial Government (South Africa); Medical Research Coordinating Committee, National Institute for Medical Research; Chief Medical Officer, Ministry of Health and Social Welfare (Tanzania). Informed written consent was obtained from the parent or guardian of each participating child on their behalf.

The study design and methods for the first two years of follow-up in the MAL-ED study have been previously described in detail [19]. Briefly, children were enrolled within the first two weeks of life and followed twice weekly through active surveillance at home visits for illnesses and feeding practices until two years of age. Anthropometry was measured monthly, and stools were collected monthly in the absence of diarrhea and whenever a diarrhea episode was identified. Enteric pathogens were detected in stool samples using quantitative PCR with the Taqman Array Card platform, as previously described [20]. *Shigella* was defined using the *ipaH* target as previously [5]. Myeloperoxidase (MPO; log [ng/ml]), a marker of neutrophil activation and infiltration, was measured in stools collected monthly in the first year of life and quarterly in the second year of life [7,21] α-1-acid glycoprotein (AGP), a marker of systemic inflammation, was measured in serum at 7, 15, and 24 months of age.

Active surveillance completed at 2 years of age, but additional follow-up visits for anthropometry and cognitive assessments were conducted at 5 years of age at all sites and at 6–8 years of age in 3 sites. At 6–8 years of age, cognitive assessments were performed to assess reasoning skills using the Raven Coloured Progressive Matrices (RCM) [22,23], and verbal fluency using a component of the NEPSY Neuropsychological Assessment [24]. Broadly, these tests were chosen to capture executive function, which is best measured once children are school-aged and is most relevant to adult functioning and human capital.

The RCM is a visual, non-language-based assessment that is applicable across languages and cultures since it tests the ability to solve a series of puzzles using analogies. RCM was chosen for children at 6–8 years because this measure was previously used among mothers in MAL-ED [25], is appropriate when access to formal schooling is limited, and is language-independent. The assessment was piloted among children who were not in the MAL-ED cohort at all three sites. RCM was acceptable and provided a range of scores, suggesting it was able to disaggregate cognitive ability in these children. The assessment was administered to study children by trained assessors in a quiet and distraction-free location.

Based on the NEPSY, semantic fluency was assessed by asking the children to name as many animals as they could in one minute, and then to name as many foods as they could in one minute. Phonemic fluency was assessed by asking them to name as many words that began with S as they could in one minute, and then to name as many words that begin with F as they could in one minute. This brief assessment has been previously used at our research site in Brazil, where childhood diarrhea burden was associated with verbal fluency [26]. This assessment was piloted at all three sites using foods, animals, and words that start with S, N, F, and P. In all languages and across sites, children generated more words that started with S and F; therefore, these letters were included in the assessments of MAL-ED study children.

### Statistical analysis

All analyses were conducted among children at the 3 sites in Haydom, Tanzania; Venda, South Africa; and Fortaleza, Brazil in which follow-up cognitive assessments were completed from 6–8 years of age.

We summarized *Shigella* burden in the first two years of life with a measure of prevalence and quantity, as done previously. [6] *Shigella* prevalence was defined as the proportion of non-diarrheal stools in which *Shigella* was detected by quantitative PCR from 0–23 months of age, and *Shigella* quantity was defined as the mean log-copy numbers per g of stool of *Shigella* detected in all non-diarrheal stools from 0–23 months. In a sensitivity analysis, we defined *Shigella* prevalence based on both non-diarrheal and diarrheal stools. To characterize intestinal inflammation, we first accounted for the observed decrease in MPO concentrations with age [21] by regressing log-MPO quantities from all measurements against a continuous variable of age in days. The mean of residuals from this model for each child was then calculated and converted to site-specific z-scores as a summarized metric of intestinal inflammation between birth to 2 years. Systemic inflammation was characterized by the mean log-AGP concentration from all available measurements at 7, 15, and 24 months of age.

Length measurements for children 2 years and younger and height measurements at 5 years of age were converted to length/height-for-age z-scores (LAZ/HAZ) using the 2006 WHO child growth standards [27]. Height measurements at 6–8 years were converted to height-for-age z-scores using the 2007 WHO Growth reference data for 5–19 years [28]. Measurements from the NEPSY were summarized into a measure of semantic fluency by summing the number of words correctly reported for animals and foods, and into a measure of phonemic fluency, by summing the number of words correctly reported that started with F and S. Both scores were converted to site-specific z-scores. Total Raven scores as a measure of reasoning skills were calculated by summing the number of correct answers in each subtest and were converted to site-specific z-scores.

We separately estimated the associations between *Shigella* burden (prevalence and quantity, defined above) and height-for-age z-score and cognitive outcomes (reasoning skills, semantic fluency, and phonemic fluency) using linear regression adjusting for site, age at the 6–8 year assessment, enrollment weight-for-age z-score (or enrollment LAZ for height outcomes), sex, socioeconomic status, exclusive breastfeeding in the first 6 months, maternal height, and the burden of each of the 12 most prevalent pathogens identified in the first 2 years of life (excluding *Shigella*). Similar models were used to estimate the associations between intestinal and systemic inflammation, defined above, and each of the outcomes. We also estimated the effects of each exposure (*Shigella* burden, *Shigella* quantity, intestinal inflammation, and systemic inflammation) on HAZ measured at 2 and 5 years of age, adjusting for the same variables except age at the 6–8 year assessment. This analysis was restricted to children who were also assessed at 6–8 years so that results could be compared across timepoints among the same children. Effects of *Shigella* prevalence, intestinal inflammation, and systemic inflammation were scaled to compare the 90^th^ percentile with the 10^th^ percentile of the observed site-specific distributions (high versus low burden), as previously [6]. Effects of *Shigella* quantity were scaled per 1 log increase in mean quantity.

Finally, we conducted a causal mediation analysis to assess whether associations between *Shigella* burden and growth and cognitive outcomes were mediated by intestinal inflammation. We used the regression-based approach by Baron and Kenny [29] using the mediation package in R [30]. We first specified a model for intestinal inflammation as the mediator with *Shigella* prevalence and all covariates included above as predictors. The outcome models included *Shigella* prevalence, intestinal inflammation, and the covariates above. We then computed the average causal mediation effect (i.e. the magnitude of the effect of *Shigella* on the outcome that is explained by *Shigella*’s effect on intestinal inflammation), the average direct effect (i.e. the magnitude of the effect of *Shigella* that is not explained by *Shigella*’s effect on intestinal inflammation), and the proportion of the total effect mediated by intestinal inflammation under these models and the sequential ignorability assumption. We conducted similar analyses by site and including *Shigella* quantity as the exposure.

## Results

We analyzed data from 451 children (73.8%) who had school-aged follow-up visits among 611 children who completed the original MAL-ED study with follow-up to 2 years of age. Specifically, we included 117 children (70.1%) in Fortaleza, Brazil, 163 (78.0%) in Haydom, Tanzania, and 171 (72.2%) in Venda, South Africa. There were no differences in sociodemographic characteristics, *Shigella* burden, inflammation, or anthropometry between the children assessed at 6–8 years of age and all children followed to 2 years (S1 Table). Children in Brazil were younger at the time of the school-aged visit (median age: 6.8 years, IQR: 6.4, 7.2) compared to those in South Africa (8.0, IQR: 7.0, 8.2) and Tanzania (7.7, IQR: 7.3, 8.0; Table 1). Of all 8 sites in the original MAL-ED study, the 3 included sites were at the extremes of the distribution of socioeconomic status. Households in Brazil and South Africa were of the highest socioeconomic status and households in Tanzania were of the lowest.

**Table 1:** Sociodemographic characteristics, burden of *Shigella* and inflammation in the first 2 years of life, and growth through 6–8 years of age among 451 children in the Brazil, South Africa, and Tanzania MAL-ED cohorts.

	Fortaleza, Brazil (n = 117)	Venda, South Africa (n = 171)	Haydom, Tanzania (n = 163)	All (n = 451)
	Median (IQR)			
**Sociodemographic characteristics**				
Age at 6–8 year assessment	6.8 (6.4, 7.2)	8.0 (7.0, 8.2)	7.7 (7.3, 8.0)	7.5 (7.0, 8.0)
Female sex (n; %)	53 (45.3)	84 (49.1)	82 (50.3)	219 (48.6)
Socioeconomic status	0.83 (0.77, 0.90)	0.80 (0.72, 0.85)	0.21 (0.14, 0.28)	0.72 (0.27, 0.84)
Percent days exclusively breastfed < 6 mo.	49 (28, 71)	17 (10, 29)	32 (19, 44)	28 (15, 46)
Maternal education (years)	9 (7, 12)	11 (9, 12)	7 (3, 7)	8 (7, 11)
Maternal height (cm)	155 (150, 160)	158 (155, 162)	156 (152, 160)	157 (152, 161)
***Shigella* burden**				
Number of non-diarrheal stools collected	20 (18, 22)	23 (22, 24)	23 (22, 24)	22 (21, 23)
Proportion positive for *Shigella*	0.05 (0, 0.07)	0.05 (0, 0.11)	0.17 (0.10, 0.23)	0.08 (0.04, 0.16)
Mean quantity of *Shigella* detected	0.21 (0, 0.4)	0.27 (0, 0.54)	1.00 (0.62, 1.42)	0.42 (0.18, 0.88)
**Intestinal inflammation**				
Number of measurements	10 (8, 11)	13 (11, 14)	12 (10, 13)	12 (10, 13)
Mean MPO concentration (log[ng/mL])	7.8 (7.4, 8.3)	8.4 (8.2, 8.7)	8.5 (8.3, 8.8)	8.4 (8.0, 8.7)
**Systemic inflammation** *				
Number of measurements	3 (2, 3)	2 (2, 3)	2 (1, 3)	2 (2, 3)
Mean AGP concentration (mg/dL)	98.5 (81.3, 117.0)	128.0 (108.7, 157.0)	115.3 (98.3, 133.6)	115.8 (96.8, 141.3)
**Anthropometry** ^†^				
Enrollment weight-for-age z-score	-0.17 (-0.77, 0.47)	-0.40 (-0.96, 0.14)	-0.02 (-0.61, 0.64)	-0.20 (-0.83, 0.43)
Enrollment length-for-age z-score	-0.72 (-1.31, -0.18)	-0.74 (-1.41, -0.18)	-0.89 (-1.59, -0.23)	-0.79 (-1.44, -0.19)
2 year length-for-age z-score	-0.03 (-0.87, 0.73)	-1.62 (-2.46, -1.05)	-2.59 (-3.22, -1.98)	-1.73 (-2.60, -0.74)
5 year height-for-age z-score	-0.08 (-0.76, 0.38)	-1.05 (-1.56, -0.40)	-1.93 (-2.53, -1.24)	-1.15 (-1.92, -0.36)
6–8 year height-for-age z-score	0.01 (-0.73, 0.69)	-0.07 (-0.69, 0.59)	-1.56 (-2.15, -1.03)	-0.58 (-1.54, 0.23)
**Cognitive outcomes** ^‡^				
Number of completed Raven tests	114	165	162	441
Number of completed NEPSY tests	113	161	161	435

* Missing for 7 children in Brazil, 5 children in South Africa, and 11 children in Tanzania

^†^Missing for 5 and 2 children at 5 and 6–8 years, respectively, in Brazil; missing for 2 and 8 children in South Africa; missing for 1 and 1 child in Tanzania.

^‡^Absolute scores not reported due to historical abuse.

The median proportion of non-diarrheal stools that were positive for *Shigella* was 17% in Tanzania compared to 5% in Brazil and South Africa. The biomarkers of intestinal and systemic inflammation were lower in Brazil compared to the other two sites. While anthropometry was similar across sites at enrollment, linear growth faltering was highly common in South Africa and Tanzania by 2 years of age, with median LAZ at 2 years of -1.62 (IQR: -2.46, -1.05) and -2.59 (IQR: -3.22, -1.98) at the two sites, respectively. While there was catch-up growth in height in South Africa (median HAZ at 6–8 years: -0.07, IQR: -0.69, 0.59), there was no catch-up growth in Tanzania (median HAZ at 6–8 years: -1.56, IQR: -2.15, -1.03; Table 1).

**Fig 1:**
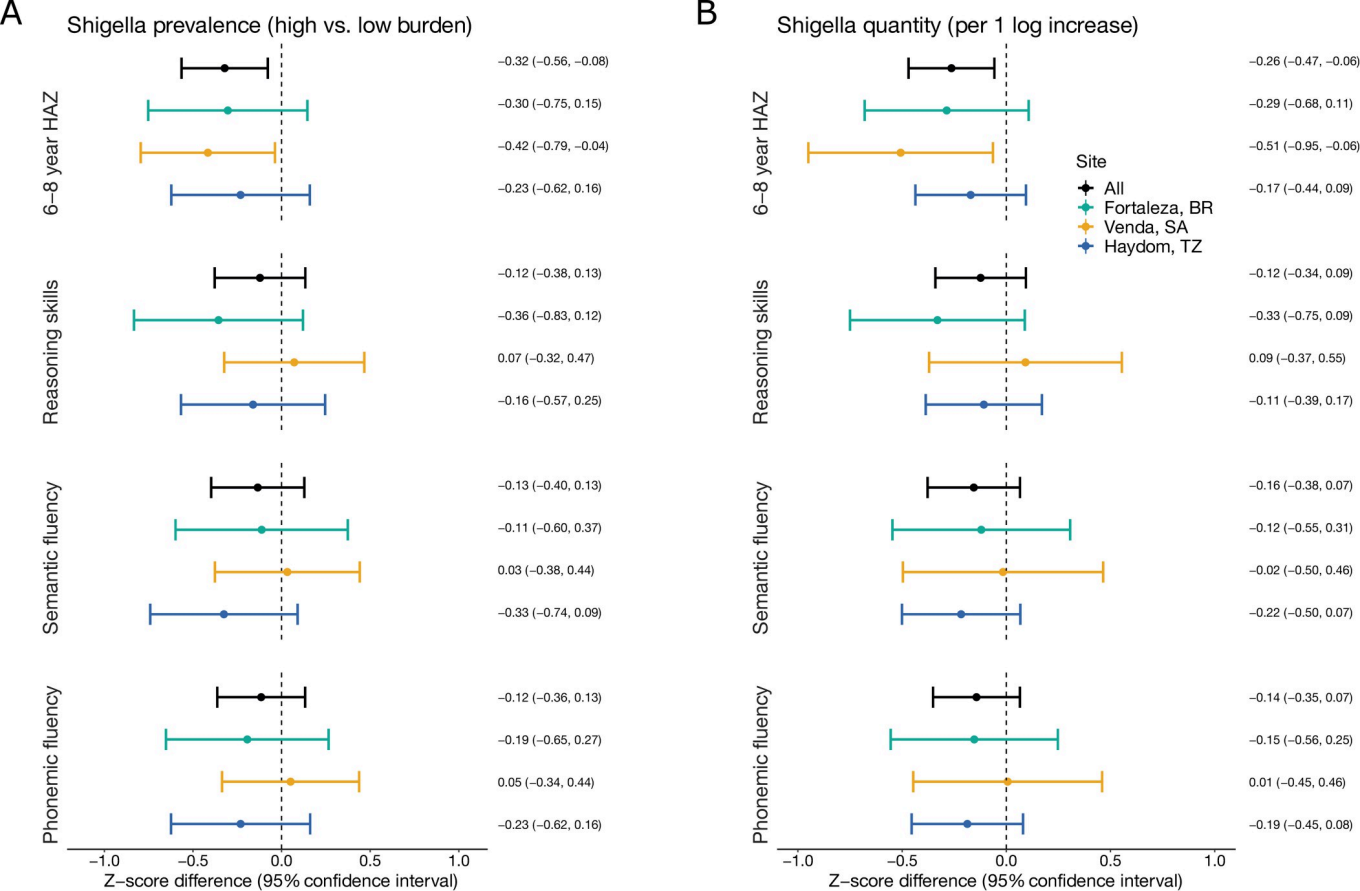
Associations between *Shigella* prevalence (A) and quantity (B) in the first 2 years of life with linear growth and cognitive outcomes at 6–8 years of age among 451 children in the Brazil, South Africa, and Tanzania MAL-ED cohorts. Mean Z-score difference estimates and 95% confidence intervals were derived from linear regression models adjusting for site, age at the 6–8 year assessment, enrollment weight-for-age z-score (or enrollment length-for-age z-score for height outcomes), sex, socioeconomic status, exclusive breastfeeding in the first 6 months, maternal height, and the burden of each of the 12 most prevalent pathogens identified in the first 2 years of life (excluding *Shigella*).

**Fig 2:**
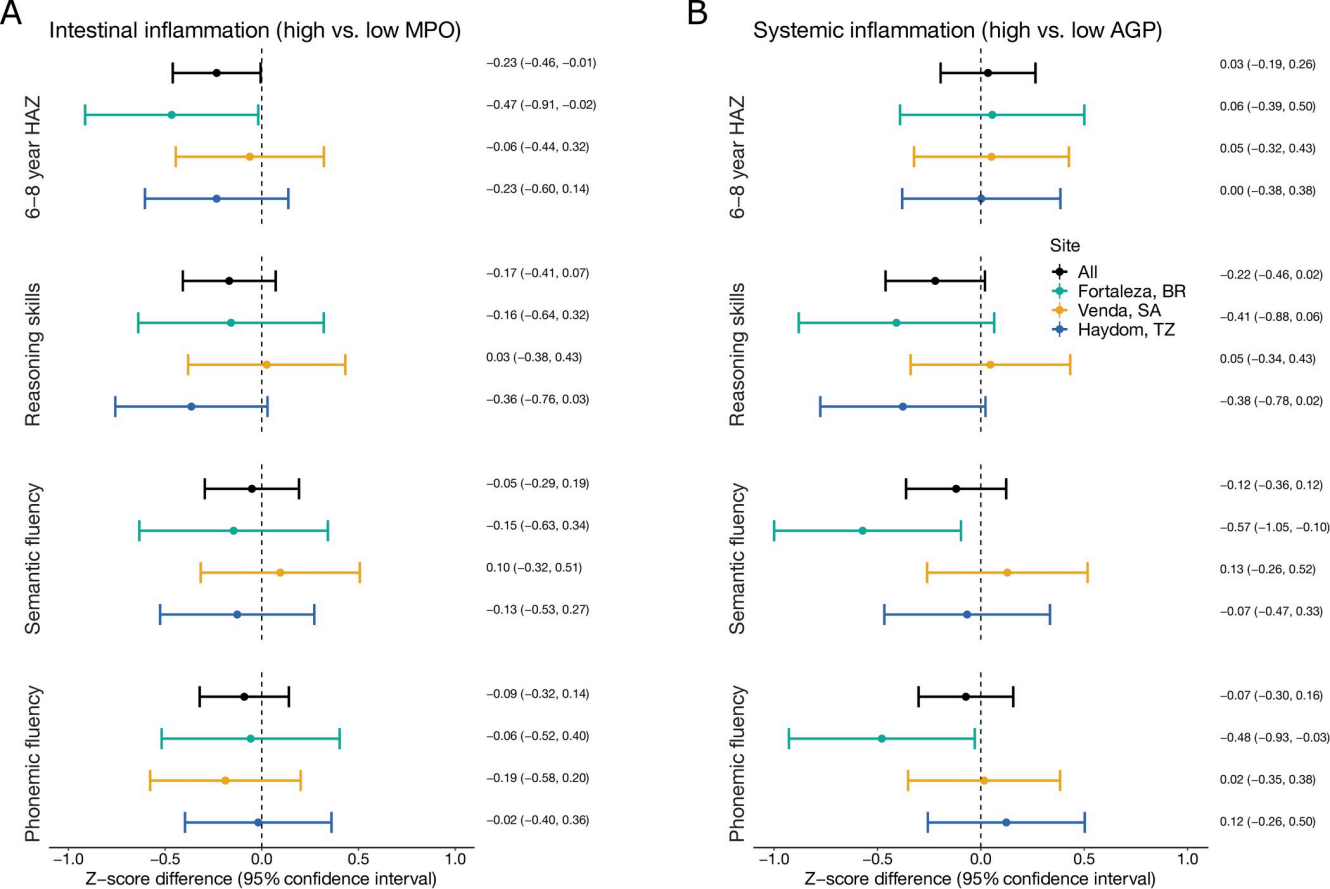
Associations between intestinal (A) and systemic (B) inflammation in the first 2 years of life with linear growth and cognitive outcomes at 6–8 years of age among 451 children in the Brazil, South Africa, and Tanzania MAL-ED cohorts. Mean Z-score difference estimates and 95% confidence intervals were derived from linear regression models adjusting for site, age at the 6–8 year assessment, enrollment weight-for-age z-score (or enrollment length-for-age z-score for height outcomes), sex, socioeconomic status, exclusive breastfeeding in the first 6 months, maternal height, and the burden of each of the 12 most prevalent pathogens identified in the first 2 years of life (excluding *Shigella*).

*Shigella* prevalence was consistently associated with decrements in height at 6–8 years (Fig 1A and S2 Table). A high prevalence of *Shigella* (at the site-specific 90^th^ percentile) was associated with a 0.32 (95% CI: 0.08, 0.56) z-score lower HAZ at 6–8 years compared to a low prevalence of *Shigella* (at the site-specific 10^th^ percentile). The association was observed in all sites, but was largest in South Africa (HAZ difference: -0.42, 95% CI: -0.79, -0.04). *Shigella* quantity was similarly associated with lower HAZ at 6–8 years, with a 0.26 (95% CI: 0.06, 0.47) z-score decrement per log increase in average quantity in non-diarrheal stools (Fig 1B and S3 Table). Intestinal inflammation had a smaller negative association with HAZ at 6–8 years (HAZ difference: -0.23, 95% CI: -0.46, -0.01), and in contrast to the association with *Shigella*, the association between inflammation and HAZ was close to the null in South Africa (Fig 2A and S4 Table). Systemic inflammation was not associated with HAZ at 6–8 years at any site (Fig 2B and S5 Table).

**Fig 3:**
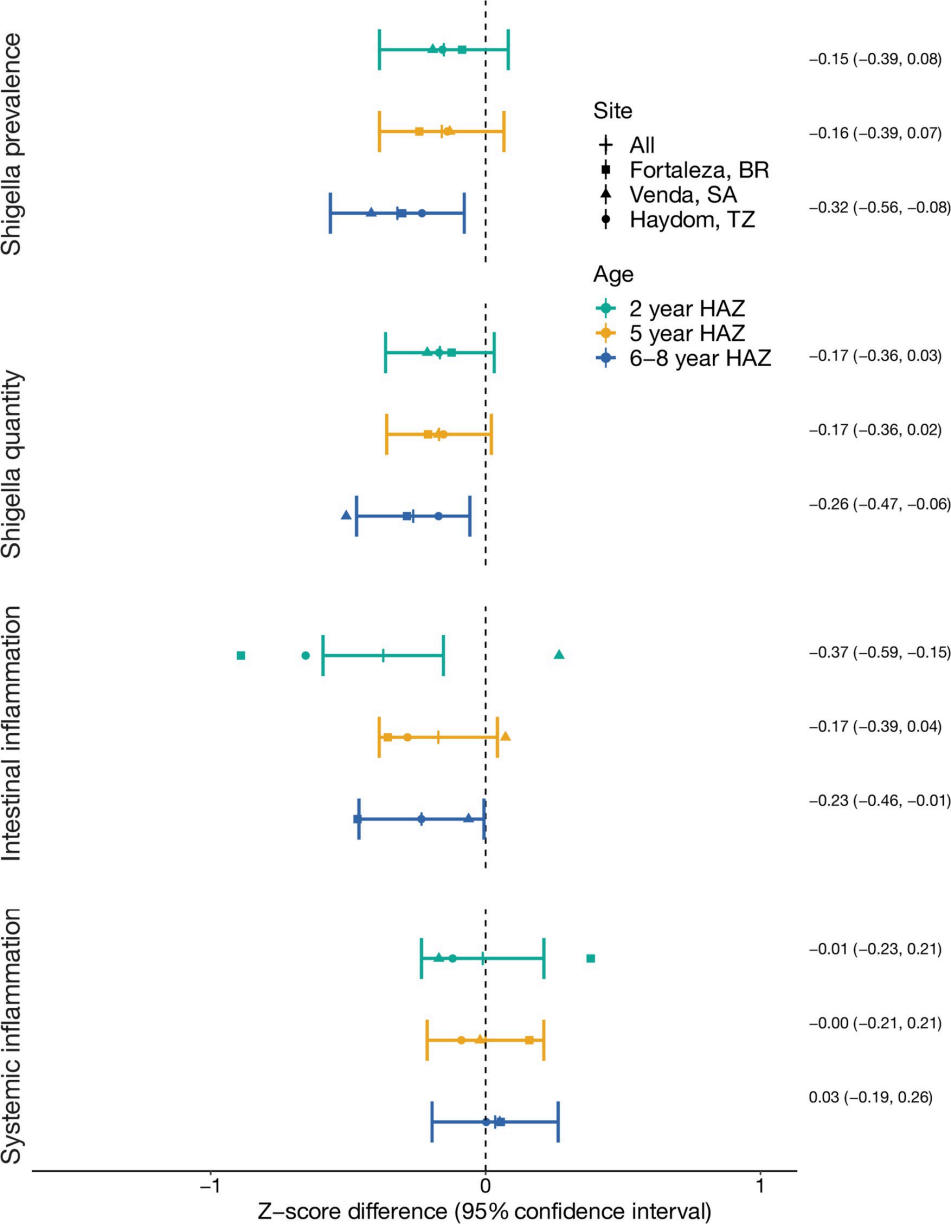
Associations between *Shigella* and inflammation in the first 2 years of life with linear growth at 2, 5, and 6–8 years of age among 451 children in the Brazil, South Africa, and Tanzania MAL-ED cohorts. Mean Z-score difference estimates and 95% confidence intervals were derived from linear regression models adjusting for site, enrollment length-for-age z-score, sex, socioeconomic status, exclusive breastfeeding in the first 6 months, maternal height, and the burden of each of the 12 most prevalent pathogens identified in the first 2 years of life (excluding *Shigella*).

The associations between *Shigella* prevalence and quantity with height at school-age were consistent with and even larger than associations with earlier height measurements at 2 and 5 years of age (Fig 3 and S2 and S3 Tables). Particularly, the associations between *Shigella* and HAZ in South Africa were larger at the oldest time point compared to 2 and 5 years. Inconsistencies across sites in the association between intestinal inflammation and height observed at 6–8 years were also observed at the earlier time points and were most extreme at 2 years of age, where high mean MPO was associated with more than half a z-score lower HAZ in Brazil and Tanzania, but higher HAZ in South Africa (LAZ difference: 0.27, 95% CI: -0.08, 0.62; S4 and S5 Tables).

The associations between *Shigella* and cognitive outcomes at 6–8 years were smaller, consistently negative in Brazil and Tanzania, and close to the null in South Africa (Fig 1 and S2 and S3 Tables). A high prevalence of *Shigella* and a 1-log increase in *Shigella* quantity were associated with between -0.1 and -0.2 z-score difference in reasoning skills, semantic fluency, and phonemic fluency scores overall. The largest site-specific effect sizes observed were modest, at approximately -0.3 z-scores, and none of the estimates were statistically significant. The associations were very similar when defining *Shigella* prevalence with both diarrheal and non-diarrheal stools.

The associations between intestinal inflammation and cognitive outcomes were mixed (Fig 2 and S4 Table). High mean MPO was associated with 0.17 (95% CI: -0.07, 0.41) z-score lower reasoning skills z-scores overall, and the association was largest in Tanzania (z-score difference: -0.36, 95% CI: -0.76, 0.03), though the estimates were not statistically significant. The associations with semantic and phonemic fluency were closer to the null and inconsistent across sites.

In contrast to its null associations with height, systemic inflammation was associated with an almost 0.4 z-score decrement in reasoning skills z-scores in Brazil and Tanzania, though again neither estimate was statistically significant. There was no corresponding association in South Africa (Fig 2 and S5 Table). Systemic inflammation was strongly associated with lower semantic and phonemic fluency scores in Brazil (semantic fluency z-score difference: -0.57, 95% CI: -1.05, -0.10; phonemic fluency z-score difference: -0.48, 95% CI: -0.93, -0.03), but was not associated with these outcomes at the other sites.

There was no evidence that intestinal inflammation mediated the association between *Shigella* and HAZ or cognitive outcomes (Table 2). The magnitude of the average causal mediation effect of *Shigella* prevalence through intestinal inflammation was close to zero for all growth and cognitive outcomes, and the proportion of the total effects of *Shigella* prevalence mediated by intestinal inflammation was less than 10% for all outcomes. The mediation effects did not differ substantially by site or when considering *Shigella* quantity as the exposure.

**Table 2:** Mediation effects to assess whether intestinal inflammation mediated the associations between *Shigella* prevalence and linear growth and cognitive outcomes at 6–8 years of age among 451 children in the Brazil, South Africa, and Tanzania MAL-ED cohorts.

	Total effect of *Shigella* prevalence	Average direct effect of *Shigella* prevalence	Average causal mediation effect of *Shigella* prevalence through intestinal inflammation	Proportion of the effect of *Shigella* prevalence mediated by intestinal inflammation
	Z-score difference (95% CI)			Proportion (95% CI)
6–8 year height-for-age z-score	-0.32 (-0.57, -0.08)	-0.31 (-0.55, -0.05)	-0.02 (-0.05, 0.00)	0.05 (-0.02, 0.26)
Reasoning skills	-0.12 (-0.36, 0.12)	-0.11 (-0.35, 0.12)	-0.01 (-0.04, 0.01)	0.09 (-0.59, 1.48)
Semantic fluency	-0.13 (-0.39, 0.13)	-0.13 (-0.39, 0.13)	-0.00 (-0.03, 0.02)	0.02 (-0.28, 0.69)
Phonemic fluency	-0.11 (-0.34, 0.13)	-0.11 (-0.33, 0.13)	-0.01 (-0.04, 0.01)	0.07 (-0.54, 0.74)

## Discussion

This study provides the first evidence of the associations between early life exposures to *Shigella*, an inflammatory pathogen, and biomarkers of inflammation on growth and cognitive outcomes at school age. A high burden of *Shigella* was associated with decrements in height at 6–8 years at all sites. We previously reported a negative association between *Shigella* and height that was sustained to 5 years of age, in contrast to diminished associations between other pathogens and height at 5 years [6]. The association between *Shigella* and height was maintained and even increased at 6–8 years. These associations corresponded to small negative associations with Raven and NEPSY cognitive test scores in Brazil and Tanzania only.

Because the presence of an association with linear growth was not necessarily predictive of an association with cognitive outcomes, anthropometric measures, and particularly height, may be limited proxies of cognitive outcomes. In Brazil, systemic inflammation was associated with lower cognitive scores, but not with linear growth faltering, which may suggest that there are pathways between early life exposures and cognitive outcomes that are independent of growth. Alternatively, since the associations with cognitive scores were all close to the null, the cognitive tests conducted in this study may not have adequately disaggregated high and low functioning children in these settings.

Given the diverse multifactorial causes of brain development, it is not surprising that direct links between early life pathogen exposures and cognition are difficult to quantify, especially in settings where resource availability is universally low and exposure to contaminated environments is high. Importantly, *Shigella* burden was driven by prevalence in non-diarrheal stools, rather than as a cause of diarrhea since 10 or fewer children in each site had *Shigella-*attributable diarrhea. *Shigella-*attributable diarrhea alone was not associated with long-term growth or cognitive outcomes in MAL-ED [31], or with linear growth and cognitive scores at 2 years of age in a cohort in Bangladesh [32].

The evidence that inflammation in the absence of illness early in life affected school-aged outcomes was also limited. There was substantial variability among associations with the biomarkers of inflammation across outcomes and sites. Intestinal inflammation was more strongly associated with height than cognitive outcomes, while systemic inflammation showed the reverse. However, the magnitudes of association were small and not statistically significant, and there was substantial heterogeneity by site, with systemic inflammation most clearly associated with poor cognitive outcomes in Brazil. These site-specific results are consistent with studies from the same location in Brazil 20 years ago, which found high diarrhea burdens were associated with deficits in verbal fluency [26,33,34].

Furthermore, despite a hypothesized causal pathway from *Shigella* to cognitive outcomes through inflammation, we found no evidence that associations between *Shigella* and outcomes were mediated by inflammation. This is apparent from the discordance in site-specific estimates between *Shigella* and inflammation on height, for example; *Shigella* had the strongest associations with decrements in HAZ at 6–8 years of age in South Africa, but inflammation was not associated with HAZ at that site. These results suggest that there may be pathways between *Shigella* and long-term outcomes that are not mediated by the pro-inflammatory properties of *Shigella*. Conversely, intestinal myeloperoxidase and serum alpha-1-acid glycoprotein measured at a few time points may inadequately describe inflammation in early life. Importantly, inflammation was not measured during symptomatic diarrhea episodes, and the effects of inflammation with diarrhea need further study.

The 3 sites included in this study represent a wide range of low-resource settings. Children in the Brazil cohort were of relatively high socioeconomic status, had low prevalence of *Shigella* and intestinal inflammation, and had little linear growth faltering. On the other extreme, children in the Tanzania cohort were of lower socioeconomic status, had a high prevalence of *Shigella* and intestinal inflammation, and a high and persistent burden of linear growth faltering. Children in South Africa were also of relatively high socioeconomic status but experienced high inflammation and had a high prevalence of stunting at 2 years of age. We appropriately accounted for heterogeneities across sites by considering site-specific z-scores, focusing on comparisons that were relevant to the observed high and low burden in each site, and estimating site-specific associations. However, the variability of results across sites highlights the limited generalizability of the determinants of growth and child development in low-resource settings. Furthermore, because the burden of *Shigella* was relatively low at two of the three sites studied, the comparisons of high-to-low burden at these sites represent small differences in these populations. Importantly, our study directly measured both cognitive outcomes and components of the hypothesized mechanism for the impact of enteric infections on child development, which improves on using height at 2 years of age as a proxy and leaving causal pathways uninterrogated. Executive function, which is most relevant for long-term human capital, is best measured when children are school aged; therefore, we were able to evaluate the long-term impact of exposures in the critical first two years of life.

A better understanding of the potential role of inflammation, local or systemic, on potential pathways between *Shigella* and growth and cognition is needed. Many markers, including CRP and pro-inflammatory cytokines, such as IL-1β, IL-6, TNF-α, IL-4, and IL-10, have been considered in addition to MPO and AGP as biomarkers of inflammation. It is unclear which marker is most predictive of outcomes and indicative of pathways related to the effects of enteropathogens. Furthermore, the most relevant timepoints for measurement are unknown. The heterogeneity in associations with inflammation across sites mirrors the variability in the characteristics of EED documented from intestinal biopsies across sites. [35] Yet another potential determinant of developmental outcomes affected by intestinal or systemic inflammation could be population genetic differences. The somewhat surprising association of ApoE4, for example, with protection against heavy diarrhea burdens and semantic fluency deficits is an example of this possibility [36].

In summary, early life exposure to *Shigella* was associated with sustained decrements in linear growth, that in some cases associated with slightly lower cognitive scores in school-aged children. While *Shigella* as a cause of inflammation may explain the impact of *Shigella* on growth, measures of MPO and AGP did not account for the associations observed in this study. Quantifying the impact and understanding mechanisms for the effect of early life exposures to enteric pathogens and EED on long-term cognitive outcomes remains challenging.

## Supporting information

S1 Table Comparison of characteristics between the 451 children assessed at 6–8 years of age and the 611 children in Brazil, South Africa, and Tanzania who completed the original MAL-ED study with follow-up to 2 years of age. (DOCX)

S2 Table Unadjusted and adjusted associations between *Shigella* prevalence in the first 2 years of life with linear growth and cognitive outcomes at 6–8 years of age among 451 children in the Brazil, South Africa, and Tanzania MAL-ED cohorts. (DOCX)

S3 Table Unadjusted and adjusted associations between *Shigella* quantity in the first 2 years of life with linear growth and cognitive outcomes at 6–8 years of age among 451 children in the Brazil, South Africa, and Tanzania MAL-ED cohorts. (DOCX)

S4 Table Unadjusted and adjusted associations between intestinal inflammation in the first 2 years of life with linear growth and cognitive outcomes at 6–8 years of age among 451 children in the Brazil, South Africa, and Tanzania MAL-ED cohorts. (DOCX)

S5 Table Unadjusted and adjusted associations between systemic inflammation in the first 2 years of life with linear growth and cognitive outcomes at 6–8 years of age among 451 children in the Brazil, South Africa, and Tanzania MAL-ED cohorts. (DOCX)
