# Supplementary material for: Impact of Shigella infections and inflammation early in life on child growth and school-aged cognitive outcomes: Findings from three birth cohorts over eight years
Source: PLoS Negl Trop Dis. 2022 Sep 23;16(9):e0010722. doi: 10.1371/journal.pntd.0010722 (PMC9534434; doi:10.1371/journal.pntd.0010722)
Supplement: S1 Table — (DOCX) [file pntd.0010722.s001.docx]

**S1 Table.** Comparison of characteristics between the 451 children assessed at 6-8 years of age and the 611 children in Brazil, South Africa, and Tanzania who completed the original MAL-ED study with follow-up to 2 years of age.

|  | Assessed at 6-8 years of age  (n=451) | Follow-up to 2 years of age  (n=611) |
| --- | --- | --- |
|  | Median (IQR) | |
| Sociodemographic characteristics |  |  |
| Female sex (n; %) | 219 (48.6) | 297 (48.6) |
| Socioeconomic status | 0.72 (0.27, 0.84) | 0.73 (0.28, 0.84) |
| Percent days exclusively breastfed < 6 mo. | 28 (15, 46) | 28 (15, 45) |
| Maternal education (years) | 8 (7, 11) | 8 (7, 11) |
| Maternal height (cm) | 157 (152, 161) | 157 (152, 161) |
| *Shigella* burden |  |  |
| Number of non-diarrheal stools collected | 22 (21, 23) | 22 (21, 23) |
| Proportion positive for *Shigella* | 0.08 (0.04, 0.16) | 0.08 (0, 0.16) |
| Mean quantity of *Shigella* detected | 0.42 (0.18, 0.88) | 0.41 (0, 0.85) |
| Intestinal inflammation |  |  |
| Number of measurements | 12 (10, 13) | 12 (10, 13) |
| Mean MPO concentration (log[ng/mL]) | 8.4 (8.0, 8.7) | 8.4 (8.0, 8.7) |
| Systemic inflammation* |  |  |
| Number of measurements | 2 (2, 3) | 2 (2, 3) |
| Mean AGP concentration (mg/dL) | 115.8 (96.8, 141.3) | 111.6 (91.8, 135.7) |
| Anthropometry |  |  |
| Enrollment weight-for-age z-score | -0.20 (-0.83, 0.43) | -0.18 (-0.80, 0.40) |
| Enrollment length-for-age z-score | -0.79 (-1.44, -0.19) | -0.81 (-1.45, -0.19) |
| 2 year length-for-age z-score | -1.73 (-2.60, -0.74) | -1.62 (-2.60, -0.61) |

*Missing for 7 children in Brazil, 5 children in South Africa, and 11 children in Tanzania

IQR = interquartile range
