# Supplementary material for: Impact of Shigella infections and inflammation early in life on child growth and school-aged cognitive outcomes: Findings from three birth cohorts over eight years
Source: PLoS Negl Trop Dis. 2022 Sep 23;16(9):e0010722. doi: 10.1371/journal.pntd.0010722 (PMC9534434; doi:10.1371/journal.pntd.0010722)
Supplement: S2 Table — (DOCX) [file pntd.0010722.s002.docx]

**S2 Table.** Unadjusted and adjusted associations between *Shigella* prevalence* in the first 2 years of life with linear growth and cognitive outcomes at 6-8 years of age among 451 children in the Brazil, South Africa, and Tanzania MAL-ED cohorts.

| Study site and outcome | Unadjusted z-score difference (95% CI) | Adjusted^†^ z-score difference (95% CI) |
| --- | --- | --- |
| All sites |  |  |
| 2 year HAZ | -0.28 (-0.52, -0.04) | -0.15 (-0.39, 0.08) |
| 5 year HAZ | -0.20 (-0.43, 0.02) | -0.16 (-0.39, 0.07) |
| 6-8 year HAZ | -0.41 (-0.64, -0.17) | -0.32 (-0.56, -0.08) |
| Reasoning skills | -0.20 (-0.44, 0.04) | -0.12 (-0.38, 0.13) |
| Semantic fluency | -0.21 (-0.45, 0.03) | -0.13 (-0.40, 0.13) |
| Phonemic fluency | -0.16 (-0.40, 0.08) | -0.12 (-0.36, 0.13) |
| Fortaleza, Brazil |  |  |
| 2 year HAZ | -0.46 (-0.93, 0.01) | -0.09 (-0.52, 0.35) |
| 5 year HAZ | -0.47 (-0.94, 0.00) | -0.24 (-0.68, 0.20) |
| 6-8 year HAZ | -0.59 (-1.05, -0.13) | -0.30 (-0.75, 0.15) |
| Reasoning skills | -0.47 (-0.93, 0.00) | -0.36 (-0.83, 0.12) |
| Semantic fluency | -0.27 (-0.74, 0.19) | -0.11 (-0.60, 0.37) |
| Phonemic fluency | -0.40 (-0.86, 0.07) | -0.19 (-0.65, 0.27) |
| Venda, South Africa |  |  |
| 2 year HAZ | -0.24 (-0.62, 0.15) | -0.19 (-0.55, 0.17) |
| 5 year HAZ | -0.09 (-0.46, 0.27) | -0.13 (-0.48, 0.21) |
| 6-8 year HAZ | -0.41 (-0.80, -0.02) | -0.42 (-0.79, -0.04) |
| Reasoning skills | -0.05 (-0.44, 0.33) | 0.07 (-0.32, 0.47) |
| Semantic fluency | -0.01 (-0.40, 0.39) | 0.03 (-0.38, 0.44) |
| Phonemic fluency | -0.06 (-0.45, 0.34) | 0.05 (-0.34, 0.44) |
| Haydom, Tanzania |  |  |
| 2 year HAZ | -0.19 (-0.59, 0.21) | -0.16 (-0.53, 0.21) |
| 5 year HAZ | -0.16 (-0.53, 0.21) | -0.14 (-0.49, 0.21) |
| 6-8 year HAZ | -0.27 (-0.66, 0.13) | -0.23 (-0.62, 0.16) |
| Reasoning skills | -0.16 (-0.55, 0.23) | -0.16 (-0.57, 0.25) |
| Semantic fluency | -0.36 (-0.76, 0.03) | -0.33 (-0.74, 0.09) |
| Phonemic fluency | -0.10 (-0.49, 0.29) | -0.23 (-0.62, 0.16) |

*Comparison of high (site-specific 90^th^ percentile) versus low (site-specific 10^th^ percentile) burden of *Shigella*

**^†^**Adjusted for site, age at the 6-8 year assessment, enrollment weight-for-age z-score (or enrollment length-for-age z-score for height outcomes), sex, socioeconomic status, exclusive breastfeeding in the first 6 months, maternal height, and the burden of each of the 12 most prevalent pathogens identified in the first 2 years of life (excluding *Shigella*).

CI = confidence interval; HAZ = height-for-age z-score
