# Supplementary material for: Impact of Shigella infections and inflammation early in life on child growth and school-aged cognitive outcomes: Findings from three birth cohorts over eight years
Source: PLoS Negl Trop Dis. 2022 Sep 23;16(9):e0010722. doi: 10.1371/journal.pntd.0010722 (PMC9534434; doi:10.1371/journal.pntd.0010722)
Supplement: S3 Table — (DOCX) [file pntd.0010722.s003.docx]

**S3 Table.** Unadjusted and adjusted associations between *Shigella* quantity in the first 2 years of life with linear growth and cognitive outcomes at 6-8 years of age among 451 children in the Brazil, South Africa, and Tanzania MAL-ED cohorts.

| Study site and outcome | Unadjusted z-score difference (95% CI) | Adjusted^†^ z-score difference (95% CI) |
| --- | --- | --- |
| All sites |  |  |
| 2 year HAZ | -0.29 (-0.49, -0.09) | -0.17 (-0.36, 0.03) |
| 5 year HAZ | -0.23 (-0.42, -0.04) | -0.17 (-0.36, 0.02) |
| 6-8 year HAZ | -0.34 (-0.54, -0.14) | -0.26 (-0.47, -0.06) |
| Reasoning skills | -0.17 (-0.36, 0.03) | -0.12 (-0.34, 0.09) |
| Semantic fluency | -0.22 (-0.42, -0.02) | -0.16 (-0.38, 0.07) |
| Phonemic fluency | -0.17 (-0.37, 0.03) | -0.14 (-0.35, 0.07) |
| Fortaleza, Brazil |  |  |
| 2 year HAZ | -0.45 (-0.86, -0.04) | -0.12 (-0.50, 0.26) |
| 5 year HAZ | -0.43 (-0.83, -0.02) | -0.21 (-0.59, 0.17) |
| 6-8 year HAZ | -0.53 (-0.93, -0.12) | -0.29 (-0.68, 0.11) |
| Reasoning skills | -0.40 (-0.81, 0.00) | -0.33 (-0.75, 0.09) |
| Semantic fluency | -0.27 (-0.68, 0.14) | -0.12 (-0.55, 0.31) |
| Phonemic fluency | -0.34 (-0.75, 0.07) | -0.15 (-0.56, 0.25) |
| Venda, South Africa |  |  |
| 2 year HAZ | -0.30 (-0.75, 0.15) | -0.21 (-0.63, 0.21) |
| 5 year HAZ | -0.17 (-0.59, 0.26) | -0.17 (-0.57, 0.23) |
| 6-8 year HAZ | -0.51 (-0.96, -0.05) | -0.51 (-0.95, -0.06) |
| Reasoning skills | -0.00 (-0.46, 0.45) | 0.09 (-0.37, 0.55) |
| Semantic fluency | -0.02 (-0.49, 0.44) | -0.02 (-0.50, 0.46) |
| Phonemic fluency | -0.14 (-0.60, 0.32) | 0.01 (-0.45, 0.46) |
| Haydom, Tanzania |  |  |
| 2 year HAZ | -0.22 (-0.48, 0.05) | -0.17 (-0.42, 0.08) |
| 5 year HAZ | -0.18 (-0.42, 0.07) | -0.15 (-0.39, 0.09) |
| 6-8 year HAZ | -0.21 (-0.47, 0.05) | -0.17 (-0.44, 0.09) |
| Reasoning skills | -0.12 (-0.38, 0.14) | -0.11 (-0.39, 0.17) |
| Semantic fluency | -0.26 (-0.52, 0.00) | -0.22 (-0.50, 0.07) |
| Phonemic fluency | -0.11 (-0.38, 0.15) | -0.19 (-0.45, 0.08) |

*Adjusted for site, age at the 6-8 year assessment, enrollment weight-for-age z-score (or enrollment length-for-age z-score for height outcomes), sex, socioeconomic status, exclusive breastfeeding in the first 6 months, maternal height, and the burden of each of the 12 most prevalent pathogens identified in the first 2 years of life (excluding *Shigella*).

CI = confidence interval; HAZ = height-for-age z-score
