# Supplementary material for: Impact of Shigella infections and inflammation early in life on child growth and school-aged cognitive outcomes: Findings from three birth cohorts over eight years
Source: PLoS Negl Trop Dis. 2022 Sep 23;16(9):e0010722. doi: 10.1371/journal.pntd.0010722 (PMC9534434; doi:10.1371/journal.pntd.0010722)
Supplement: S4 Table — (DOCX) [file pntd.0010722.s004.docx]

**S4 Table.** Unadjusted and adjusted associations between intestinal inflammation* in the first 2 years of life with linear growth and cognitive outcomes at 6-8 years of age among 451 children in the Brazil, South Africa, and Tanzania MAL-ED cohorts.

| Study site and outcome | Unadjusted z-score difference (95% CI) | Adjusted^†^ z-score difference (95% CI) |
| --- | --- | --- |
| All sites |  |  |
| 2 year HAZ | -0.43 (-0.67, -0.20) | -0.37 (-0.59, -0.15) |
| 5 year HAZ | -0.27 (-0.50, -0.05) | -0.17 (-0.39, 0.04) |
| 6-8 year HAZ | -0.33 (-0.57, -0.09) | -0.23 (-0.46, -0.01) |
| Reasoning skills | -0.20 (-0.43, 0.04) | -0.17 (-0.41, 0.07) |
| Semantic fluency | -0.08 (-0.32, 0.16) | -0.05 (-0.29, 0.19) |
| Phonemic fluency | -0.10 (-0.33, 0.14) | -0.09 (-0.32, 0.14) |
| Fortaleza, Brazil |  |  |
| 2 year HAZ | -1.00 (-1.46, -0.54) | -0.89 (-1.30, -0.48) |
| 5 year HAZ | -0.58 (-1.03, -0.14) | -0.36 (-0.77, 0.06) |
| 6-8 year HAZ | -0.52 (-0.99, -0.06) | -0.47 (-0.91, -0.02) |
| Reasoning skills | -0.35 (-0.81, 0.12) | -0.16 (-0.64, 0.32) |
| Semantic fluency | -0.20 (-0.67, 0.26) | -0.15 (-0.63, 0.34) |
| Phonemic fluency | -0.38 (-0.84, 0.09) | -0.06 (-0.52, 0.40) |
| Venda, South Africa |  |  |
| 2 year HAZ | 0.13 (-0.25, 0.51) | 0.27 (-0.08, 0.62) |
| 5 year HAZ | -0.04 (-0.4, 0.32) | 0.07 (-0.28, 0.42) |
| 6-8 year HAZ | -0.27 (-0.66, 0.11) | -0.06 (-0.44, 0.32) |
| Reasoning skills | 0.05 (-0.34, 0.43) | 0.03 (-0.38, 0.43) |
| Semantic fluency | 0.01 (-0.38, 0.40) | 0.10 (-0.32, 0.51) |
| Phonemic fluency | -0.03 (-0.42, 0.36) | -0.19 (-0.58, 0.20) |
| Haydom, Tanzania |  |  |
| 2 year HAZ | -0.63 (-1.01, -0.24) | -0.65 (-1.01, -0.30) |
| 5 year HAZ | -0.30 (-0.68, 0.07) | -0.28 (-0.63, 0.07) |
| 6-8 year HAZ | -0.24 (-0.64, 0.15) | -0.23 (-0.60, 0.14) |
| Reasoning skills | -0.34 (-0.73, 0.05) | -0.36 (-0.76, 0.03) |
| Semantic fluency | -0.09 (-0.48, 0.31) | -0.13 (-0.53, 0.27) |
| Phonemic fluency | 0.04 (-0.35, 0.44) | -0.02 (-0.40, 0.36) |

*Comparison of high (site-specific 90^th^ percentile) versus low (site-specific 10^th^ percentile) levels of fecal myeloperoxidase

**^†^**Adjusted for site, age at the 6-8 year assessment, enrollment weight-for-age z-score (or enrollment length-for-age z-score for height outcomes), sex, socioeconomic status, exclusive breastfeeding in the first 6 months, maternal height, and the burden of each of the 12 most prevalent pathogens identified in the first 2 years of life (excluding *Shigella*).

CI = confidence interval; HAZ = height-for-age z-score
