# Supplementary material for: Impact of Shigella infections and inflammation early in life on child growth and school-aged cognitive outcomes: Findings from three birth cohorts over eight years
Source: PLoS Negl Trop Dis. 2022 Sep 23;16(9):e0010722. doi: 10.1371/journal.pntd.0010722 (PMC9534434; doi:10.1371/journal.pntd.0010722)
Supplement: S5 Table — (DOCX) [file pntd.0010722.s005.docx]

**S5 Table.** Unadjusted and adjusted associations between systemic inflammation* in the first 2 years of life with linear growth and cognitive outcomes at 6-8 years of age among 451 children in the Brazil, South Africa, and Tanzania MAL-ED cohorts.

| Study site and outcome | Unadjusted z-score difference (95% CI) | Adjusted^†^ z-score difference (95% CI) |
| --- | --- | --- |
| All sites |  |  |
| 2 year HAZ | -0.03 (-0.27, 0.22) | -0.01 (-0.23, 0.21) |
| 5 year HAZ | -0.01 (-0.24, 0.21) | -0.00 (-0.21, 0.21) |
| 6-8 year HAZ | -0.01 (-0.26, 0.23) | 0.03 (-0.19, 0.26) |
| Reasoning skills | -0.22 (-0.46, 0.02) | -0.22 (-0.46, 0.02) |
| Semantic fluency | -0.11 (-0.35, 0.13) | -0.12 (-0.36, 0.12) |
| Phonemic fluency | -0.08 (-0.32, 0.16) | -0.07 (-0.30, 0.16) |
| Fortaleza, Brazil |  |  |
| 2 year HAZ | 0.33 (-0.15, 0.81) | 0.38 (-0.05, 0.82) |
| 5 year HAZ | 0.06 (-0.40, 0.52) | 0.16 (-0.27, 0.58) |
| 6-8 year HAZ | -0.00 (-0.48, 0.47) | 0.06 (-0.39, 0.50) |
| Reasoning skills | -0.48 (-0.96, -0.01) | -0.41 (-0.88, 0.06) |
| Semantic fluency | -0.59 (-1.06, -0.11) | -0.57 (-1.05, -0.10) |
| Phonemic fluency | -0.56 (-1.03, -0.09) | -0.48 (-0.93, -0.03) |
| Venda, South Africa |  |  |
| 2 year HAZ | -0.01 (-0.40, 0.38) | -0.17 (-0.53, 0.19) |
| 5 year HAZ | 0.12 (-0.24, 0.49) | -0.02 (-0.36, 0.32) |
| 6-8 year HAZ | 0.13 (-0.26, 0.53) | 0.05 (-0.32, 0.43) |
| Reasoning skills | 0.11 (-0.28, 0.50) | 0.05 (-0.34, 0.43) |
| Semantic fluency | 0.15 (-0.24, 0.54) | 0.13 (-0.26, 0.52) |
| Phonemic fluency | 0.09 (-0.29, 0.48) | 0.02 (-0.35, 0.38) |
| Haydom, Tanzania |  |  |
| 2 year HAZ | -0.30 (-0.71, 0.10) | -0.12 (-0.49, 0.25) |
| 5 year HAZ | -0.21 (-0.59, 0.16) | -0.09 (-0.44, 0.26) |
| 6-8 year HAZ | -0.17 (-0.57, 0.23) | 0.00 (-0.38, 0.38) |
| Reasoning skills | -0.37 (-0.77, 0.03) | -0.38 (-0.78, 0.02) |
| Semantic fluency | -0.06 (-0.45, 0.34) | -0.07 (-0.47, 0.33) |
| Phonemic fluency | 0.07 (-0.32, 0.47) | 0.12 (-0.26, 0.50) |

*Comparison of high (site-specific 90^th^ percentile) versus low (site-specific 10^th^ percentile) levels of serum alpha-1-acid glycoprotein

**^†^**Adjusted for site, age at the 6-8 year assessment, enrollment weight-for-age z-score (or enrollment length-for-age z-score for height outcomes), sex, socioeconomic status, exclusive breastfeeding in the first 6 months, maternal height, and the burden of each of the 12 most prevalent pathogens identified in the first 2 years of life (excluding *Shigella*).

CI = confidence interval; HAZ = height-for-age z-score
